# Differentiating Psychosomatic, Somatopsychic, Multisystem Illnesses and Medical Uncertainty

**DOI:** 10.3390/healthcare7040114

**Published:** 2019-10-08

**Authors:** Robert C. Bransfield, Kenneth J. Friedman

**Affiliations:** 1 Department of Psychiatry, Rutgers-Robert Wood Johnson Medical School, Piscataway, NJ 08854, USA; 2 Pharmacology and Physiology, NJ Medical School, Newark, NJ 07103, USA; kenneth.j.friedman@gmail.com

**Keywords:** psychosomatic, somatopsychic, multisystem illness, medical uncertainty, Lyme disease, Morgellons disease, psychogenic, bodily distress, myalgic encephalitis/chronic fatigue syndrome

## Abstract

There is often difficulty differentiating between psychosomatic, somatopsychic, multisystem illness, and different degrees of medical uncertainty. Uncommon, complex, and multisystem diseases are commonly misdiagnosed. Two case histories are described, and relevant terms differentiating psychosomatic, somatopsychic, and multisystem illnesses are identified, reviewed, and discussed. Adequate differentiation requires an understanding of the mind/body connection, which includes knowledge of general medicine, psychiatry, and the systems linking the body and the brain. A psychiatric diagnosis cannot be given solely based upon the absence of physical, laboratory, or pathological findings. Medically unexplained symptoms, somatoform disorder, and compensation neurosis are outdated and/or inaccurate terms. The terms subjective, nonspecific, and vague can be used inaccurately. Conversion disorders, functional disorders, psychogenic illness, factitious disorder imposed upon another (Munchausen’s syndrome by proxy), somatic symptom disorder, psychogenic seizures, psychogenic pain, psychogenic fatigue, and delusional parasitosis can be over-diagnosed. Bodily distress disorder and bodily distress syndrome are scientifically unsupported and inaccurate. Many “all in your head” conditions may be related to the microbiome and the immune system. Better education concerning the interface between medicine and psychiatry and the associated diagnostic nomenclature as well as utilizing clinical judgment and thorough assessment, exercising humility, and maintaining our roots in traditional medicine will help to improve diagnostic accuracy and patient trust.

## 1. Introduction

### 1.1. Gaps, Restrictiveness, and Deficiencies in the Healthcare Systems

Many physicians find it challenging when making a diagnosis involving the interface between general medical and psychiatric illnesses, and diagnostic errors harm patients. Historically, there has been a bias in which poorly understood illnesses are often considered to have a psychiatric origin until the pathophysiology is better understood and explained on some other basis. There is a broad spectrum of opinion regarding how to approach a diagnosis when there is a general medical and psychiatric differential diagnosis [1]. 

In recent years, medicine has gravitated towards a pressure to comply with third party guidelines and computerized algorithms, and there has been a trend towards super specialization with limited training in non-specialty fields. The combination of these issues has collectively contributed to a silo mentality and a fragmentation of knowledge [2]. Restrictive, third-party guidelines, time constraints, and restrictive, computerized algorithms have often limited the thoroughness of a physician’s evaluation. In one study, in 67% of encounters in which clinicians elicited patient concerns, the clinician interrupted the patient after a median of only 11 seconds [3]. These limitations impede the adequacy of the clinical assessment of complex diseases.

Knowledge gaps can be associated with several issues: (1) a lack of knowledge in either psychiatry or medicine, (2) gaps between clinical expertise and research knowledge, (3) difficulties converting research results derived from groups of subjects to the uniqueness of individual patients, and (4) the improper application of guidelines. 

The average physician who practices in an internal medicine specialty, including many who write guidelines that others follow, may have a very limited basic background in psychiatry, often consisting of a one-month rotation through a state hospital while in medical school, and little continuing medical education in psychiatry or psychosomatic medicine since then. Although psychiatrists have a strong foundation in general medicine, not all psychiatrists keep current in general medicine, and many other mental health professionals have very limited training in general medicine. With specialization and fragmentation in healthcare systems, there are many healthcare providers lacking capability in both psychiatry and general medicine. As a result, the understanding of the interface between mental and somatic disorders falls into a gap between psychiatry and medicine. It is a concern when thought leaders lacking adequate knowledge in both psychiatry and general medicine write and promote diagnostic and treatment guidelines that others may then follow rigidly on subjects such as somatoform disorder, somatic symptom disorder, medically unexplained symptoms, bodily distress disorder, and multisystem illnesses. 

Another gap exists between clinical expertise and medical research. This can result in difficulty reconciling differences between clinical observation and clinician experience vs. research performed by bench scientists and academicians who often have limited clinical capabilities. This is referred to as translational research, in which there is a unidirectional continuum where research findings are moved from the researcher’s bench to the patient’s bedside [4]. However, more effective progress is achieved when there is also a bidirectional process in which clinical observations and wisdom also inform basic science research.

Many who write United States Centers for Diseases Control and Prevention’s guidelines are epidemiologists, microbiologists, and other researchers rather than practicing clinicians who have the long-term responsibility for treating patients. For example, the Second National Conference on Serological diagnosis of Lyme Disease in Dearborn, Michigan, USA in 1994 resulted in the Association of State and Territorial Public Health Laboratory Director’s (ASTPHLD)’s criteria for the diagnosis of Lyme disease. Most who attended this meeting were not physicians, and the few physicians who attended were academicians and researchers rather than physicians practicing in the community which had higher levels of clinical experience and expertise [5].

Another limitation occurs when research is performed on groups of individuals, which generate statistics for groups of patients that are then translated into a specific treatment for the individual patient. Patients with unique presentations are being compromised by an emphasis upon population-based standards of care rather than their individual patient needs and experiences. It is far better for physicians to rely less upon clinical guidelines based upon group statistics for managing single diseases and instead rely more on their own clinical judgment to create treatment plans that are tailored to meet the needs of individual patients [6]. Diseases involving brain and body interaction are particularly challenging. In view of the uniqueness of individuals, biological heterogeneity, the complexity of some illnesses, and individual differences in treatment tolerance, safety, and efficacy, any treatment based upon rigid adherence to treatment guidelines derived from groups and applied to individual patients without exercising clinical judgment is clearly below the standard of care [7,8,9]. As a result of these multiple issues, patients with complex illnesses can feel lost and abandoned by the healthcare system.

### 1.2. Consequences of Diagnostic Errors

Complex brain–body differential diagnoses are challenging to payers, physicians, and affected patients. 

Patients often describe going to many different physicians before they acquire an accurate diagnosis. One survey of over 12,000 participants found the average patient with Lyme disease was seen by five physicians before they were properly diagnosed [10]. When the time allowed for a more thorough assessment is limited by healthcare reimbursement policies, physicians often respond by ordering an excessive amount of testing. Healthcare financial resources are strained, since these patients may not fit well into current diagnostic and treatment algorithms, and the evaluations of these patients may result in multiple tests and consultations of limited cost effectiveness [11]. Diagnostic delays also result in increased costs from disability, lost productivity, and caretaker burden. The payers who assume these financial burdens may include insurance companies, government healthcare systems, employers, patients, and patient’s families. Many insurance companies have barriers and limitations of what they cover, and patients with complex, chronic, and costly diseases incur significant out-of-pocket costs. A significant number of medical bankruptcies occur among both insured and uninsured individuals [12,13]. When dealing with these challenging cases, some physicians view these patients as being difficult, frustrating, and demanding [14]. In addition, some physicians react to these difficult cases by becoming highly stressed [15]. However, the greatest stress is endured by the patients who report feeling dissatisfied, disbelieved, and dismissed by clinicians [16].

Brain–body diagnostic errors are common in these patients, and these errors receive considerable attention in both the media and in the medical literature. Most of the cases receive this attention in books, media, and journal articles and involve erroneous psychiatric diagnoses rather than medical diagnoses [17,18].

Females are more often given an incorrect psychosomatic diagnosis, indicating gender-based bias and lack of research/understanding on how the female body responds to biological illness [19]. A recent book explored the systemic problems of women’s experiences of being dismissed by the medical providers. This included being discharged from a hospital emergency department mid-heart attack with a prescription for anti-anxiety meds, having autoimmune diseases and being labeled “chronic complainers” for years before being properly diagnosed, and having endometriosis and being told they are just overreacting to “normal” menstrual cramps [19]. Illnesses such as chronic fatigue syndrome and fibromyalgia are “contested” illnesses. They are considered psychosomatic and not “real” illnesses. They are given labels such as “hysteria”, “hypochondriacal”, or “all in their head” [19]. 

There are many reported cases of misdiagnosis and treatment delays in the media. One case that drew considerable media attention was the case of Julia, who was in a wheelchair from Lyme disease and was blessed by the Pope when he visited Philadelphia. Two psychiatrists independently cleared Julia of any psychological cause for her symptoms. The attending pediatrician refused to accept either report. To rule out her suspicion of malingering, she had the physical therapist purposely drop Julia on the concrete floor [20,21,22]. Another case drawing considerable attention was a woman in the United Kingdom who was told her symptoms were “all in her head” and was diagnosed with somatization disorder, which resulted in a 20-year treatment delay [23]. 

An example of misdiagnosis reported in the medical literature is the case of a 57-year-old woman with pain and discomfort in multiple sites on her upper body. She was diagnosed with somatic symptom disorder after a partial examination turned out to be negative. Falsely diagnosed as having somatic symptom disorder for six months, she was then correctly diagnosed as having multiple myeloma [24]. Another example of diagnostic errors and improper treatment is gastroenterological patients whose symptoms were of unclear etiology and were most commonly treated with antipsychotics [25].

One interesting study found in the Dutch General Practice Registry showed patients with a diagnosis of somatoform disorders had a higher infection load compared to matched controls preceding their diagnosis. The results of the study demonstrated a somatopsychic process. However, the authors concluded the opposite by stating the infection caused somatoform disorders, which instead would be viewed as psychological symptoms causing physical symptoms [26]. Other similar studies conclude a strong causal association between infections and psychiatric illness [27,28]. 

Difficult-to-diagnose cases are often viewed as invisible illnesses, since there may be no outward appearance of illness by a superficial examination. Many people suffering from these chronic, invisible illnesses such as myalgic encephalomyeletis/chronic fatigue syndrome, fibromyalgia, Lyme disease, and postural orthostatic tachycardia syndrome (POTS) are frequently misdiagnosed. They are tired of being unheard and told symptoms are imaginary, self-inflicted, and psychosomatic. As a result of this, they often describe feelings of abandonment from physicians and the healthcare system, which results in increased risks of suicidal ideation, suicide attempts, and suicide compared with the general population [29].

### 1.3. Guidelines

All guidelines have limitations and disclaimers that individualized judgment is necessary. Different guidelines have different levels of reliance upon randomized, controlled studies, which have two major limitations: (1) Once a certain level of knowledge is achieved by doing these studies, it is no longer ethical to continue further placebo-controlled studies. (2) Any given research may not be relevant to the uniqueness of any particular individual. Because of these and other limitations, guidelines are useful but cannot be universally applied [30].

Flawed guidelines have resulted from flawed research and bias. This problem is further compounded when well-intentioned physicians follow these guidelines assuming they are trustworthy. Examples of this have occurred with myalgic encephalomyelitis/chronic fatigue syndrome, Lyme disease, and “medically unexplained symptoms.” 

A lack of understanding of myalgic encephalomyelitis/chronic fatigue syndrome both prior to and after the Institute of Medicine Report on this disorder has contributed to many patients feeling maligned, blamed, untreated, and undertreated. Some patients stated that they felt belittled, dismissed, and ignored by their health care professionals who followed some of the commonly disseminated guidelines. More than 80% of patients with myalgic encephalomyelitis/chronic fatigue syndrome (CFS) go undiagnosed, while 65% of patients spend more than a year seeking the correct diagnosis [31,32,33,34]. Treatment recommendations based upon a graded activity and a cognitive behavioral therapy (PACE) trial were previously adopted by many healthcare organizations. However, the research was highly flawed and never supported the belief that ignoring symptoms would lead to recovery [35,36]. The inaccurate treatment recommendations based upon the PACE trial recommended patients should ignore symptoms. In addition, patients were given a form of cognitive behavior therapy that challenged their beliefs of their having any physiological illness limiting their ability to exercise. Instead, to become more active—and possibly fully recover—they only needed to ignore their symptoms [37]. Many patients failed to respond to this treatment, and the research supporting the concept that exercise can treat chronic fatigue syndrome was subsequently rejected by Cochrane stating that the work does not meet the organization’s “quality standards.” [38]. After the PACE study was found to be invalid, there have been further advances in the field, and many guidelines have since been revised [39].

Another set of guidelines that failed to adequately address brain–body interactions is The Clinical Assessment, Treatment, and Prevention of Lyme Disease, Human Granulocytic Anaplasmosis, and Babesiosis: Clinical Practice Guidelines by the Infectious Diseases Society of America (IDSA) [40]. These guidelines gave excessive credibility to flawed testing, failed to recognize the psychiatric symptoms caused by Lyme and other tick-borne diseases, and instead discounted many of the late stage symptoms as being ”the aches and pains of daily living”, subjective and non-specific, and medically unexplained symptoms. The IDSA Lyme disease guidelines have had multiple criticisms, including being highly biased and lacking objectivity, since the day they were published [41,42,43,44,45,46,47]. The Institute of Medicine’s report, Clinical Practice Guidelines We Can Trust [48], uses the example of the IDSA Lyme disease guidelines to illustrate issues associated with untrustworthiness, including problems of conflicts of interests, lack of transparency, and scientific bias in guidelines development—“Unfortunately, patients cannot put their chronic illness on hold until the medical scientists come to a consensus on whether the evidence suggesting infectious causation is or is not close enough to ‘definitive.’ Making wise decisions in an uncertain environment requires balanced reasoning, critical thinking, compassion, and common sense… Some players in the Lyme controversy seem to pride themselves in their acceptance of a conclusion only when the evidence overwhelmingly supports it…” [49]. The IDSA is in the process of revising these guidelines in 2019, and a draft of its guidelines was posted briefly for public comment. The revised guidelines showed little change in most of the areas of controversy, and attention to some of the flaws in the proposed *guidelines has been addressed* [50,51,52].

*A guideline called Medically Unexplained Symptoms (MUS) in Children and Young People: A Guide to Assessing and Managing Patients Under the Age of 18 Who Are Referred to Secondary Care* was endorsed by the Royal College of Psychiatrists and the Paediatric Mental Health Association and gives a clear appearance of a bias to benefit third parties and physicians rather than patients [53]. In this guideline, the authors attempt to revive the concept of medically unexplained symptoms (MUS), even though it has been considered an invalid concept since the 2013 publication of the 5th edition of the American Psychiatric Association’s (APA) *Diagnostic and Statistical Manual of Mental disorders 5th Edition* (DSM-5) [54]. This guideline encouraged doctors to consider a diagnosis of MUS if: “(1) Your patient has undergone an unusual level of investigations and/or been to a significant number of hospital specialists relative to their diagnosis. (2) You experience a high level of anxiety when seeing the patient and their family, and/or feel pressured into referring for investigations or to other specialists in a way that you don’t experience with other patients in a similar clinic setting. (3) You feel irritated with the patient or their family for not ‘getting better.’ (4) There is a family history of MUS. (5) There is significant absence from school as a result of symptoms that appear ‘out of proportion’ to physical investigations. (6) You have an experience of a parent who appears overly-invested in their child’s illness and loss of function” [53].

## 2. Materials and Methods 

Two case presentations are given to demonstrate some of the relevant issues when differentiating between psychosomatic, somatopsychic, and multisystem illnesses, and they are discussed herein. The patients’ identities are concealed, and written consent for publication was acquired. To help make a differential diagnosis, relevant brain–body diagnostic terms used in making a differential diagnosis and terms in which there is confusion, controversy, debate, misdiagnoses, and abuse are then identified. Some of these are terms the first author has encountered while doing consultation-liaison psychiatry. Additional terms are considered for inclusion using searches of PubMed, Google Scholar, and the author’s archives. The terms are then defined and discussed. Some of these terms are identified and defined in formalized diagnostic references, which include the APA DSM-5 [54] and the *International Classification of Diseases* (ICD), which are considered standards for diagnosis [54,55]. Other terms may be included in only one or neither of these references. The terms that are defined include APA DSM-5 diagnostic categories, mental health, mental illness, psychosomatic disorders, somatopsychic disorders, multisystem disorders, medical uncertainty, somatoform disorders, medically unexplained symptoms, functional disorders, psychogenic disorders, compensation neurosis, psychogenic seizures, psychogenic pain, psychogenic fatigue, delusional parasitosis, subjective vs. objective, non-specific and vague symptoms, bodily distress disorder, and bodily distress syndrome. Relevant terms in the DSM-5 are followed by the DSM-5 diagnostic code with the associated *International Classification of Diseases* (ICD) codes following in parentheses [54,55]. Articles relevant to defining and differentiating psychosomatic, somatopsychic, and multisystem illnesses are reviewed. Terms with the greatest potential for misuse and abuse are discussed in greater detail. Significant issues relevant to accurate diagnosis and diagnostic errors are discussed. Conclusions are drawn to aid the clinician in differentiating psychosomatic, somatopsychic, multisystem illnesses, and medical uncertainty.

## 3. Results

### 3.1. Case Presentations and Discussion

#### 3.1.1. Case Presentations

Patient A is an 18-year-old white female with multiple symptoms who had previously been healthy and adept at Taekwondo. She had a bull’s eye rash followed by a Bell’s palsy; she became increasingly debilitated over four years and subsequently needed a wheelchair. She had seizure episodes. Prior diagnoses included “wanting attention”, fibromyalgia, chronic fatigue, hypoglycemia, and pseudoseizures. The major symptoms included cognitive impairments (attention, memory, processing speed, concentration/executive functioning), tactile hypersensitivity, sun sensitivity, orthostatic hypotension, weight loss, fatigue, non-restorative sleep, pelvic pain, difficulty urinating, headaches peripheral neuropathy, muscle atrophy, cervical radiculopathy, hair loss, costochondritis, subluxation of multiple joints, and generalized pain. After more thorough assessments, the eventual diagnosis was late stage Lyme borreliosis with multisystem symptoms, porphyria, Ehlers-Danlos/ALPIM syndrome (anxiety-laxity-pain-immune-mood) [56] with seizures caused by increased intracranial pressure from cranio-cervical instability. On closer evaluation, the patient had complex partial seizures and did not have “pseudoseizures”. The patient was subsequently treated and is now physically active, married, and leading a productive life.

Patient B lives in England and was diagnosed with reactive arthritis causing leg pain when she was 12 years old. She then developed an excruciating headache accompanied by a complete loss of balance and involuntary jerking movements, which resulted in her mother bringing her to the hospital where she was admitted for one night. She was brought back to the hospital daily for several days as increasing and intensifying symptoms developed. The first doctor to assess her wrote, “Hysteria, possible conversion disorder” in her notes. Following this impression, no relevant investigations were performed. Patient B was left deteriorating and untreated, by which time she was having constant seizures and needed a wheelchair. Her mother repeatedly told them that Lyme disease was highly suspected since the family lived in a region known to be epidemic for Lyme disease and other relatives had been diagnosed with the disease and begged them to help her daughter. These appeals were ignored. Her mother took Patient B to a private clinic where a consultant thoroughly examined her and diagnosed encephalitis and possible encephalomyelitis (inflammation of the brain/brainstem/spinal cord), probably due to Lyme disease. She was immediately put on intravenous antibiotics at the clinic for four days. In 36 hours, the seizures had stopped, and her headache slowly improved. Her blood tests came back positive for Lyme disease. The hospital admitted their error and gave an unreserved apology. On instructions from the consultant, Patient B had a further three months of daily intravenous antibiotic treatment at a National Health Service Hospital. After about two months, Patient B was able to walk again, but when the antibiotics were stopped, the seizures and other symptoms returned. The family raised funds to take Patient B to the United States for treatment by a physician who had experience with such cases. The treatment stabilized her condition and brought great improvement to some of her symptoms. However, due to the treatment delay, she still had some persistent health issues, including severe headaches, joint pains, extreme fatigue, cognitive dysfunction, and other symptoms. 

#### 3.1.2. Discussion of Case Presentations

In both cases, the complexity of a multisystem illness was not understood nor adequately pursued by the treating physicians. The patients’ symptoms were conceptualized as being caused by the onset of a psychiatric illness that was given as a diagnosis by default, such as wanting attention, pseudoseizures, hysteria, and possible conversion disorder, even though there was no adequate psychiatric assessment and no valid psychodynamic basis to support such a diagnosis. This led to tragic delays in diagnosis and treatment to both patients. The first author has seen and published descriptions of many other tragic cases in his practice [57,58,59,60,61,62].

### 3.2. Defining Relevant Terms

When dealing with complex, inadequately investigated conditions in which many symptoms identified on a thorough history and review of systems are insufficiently or wholly unsupported by commonly used clinical laboratory tests, it is best to begin with definitions. Socrates stated, “The beginning of wisdom is the definition of terms” [63]. The symptoms expressed by these patients suggest a mind–body interplay; therefore, it is important to define terms that are most relevant to this, when making a diagnosis. These terms include mental health and mental illness, psychosomatic, somatopsychic, multisystem illness, medical uncertainty, and DSM-5, ICD, and other terms [54,55]. 

The American Psychiatric Association *Diagnostic and Statistical Manual of Mental Disorders* was first developed in 1952. It was a variant of the 1948 6th Revision of the International Lists of Diseases and Causes of Death. It expanded upon descriptions of psychiatric diagnostic categories and was the first official manual of mental disorders to focus on clinical use. Since then, the two different diagnostic systems have evolved through different but sometimes related processes. The current *International Classification of Disease* is the 10th Revision; however, the proposed 11^th^ Revision will soon be implemented [55].

#### 3.2.1. Mental Health

Mental health is not defined in the APA DSM-5 or in the ICD, and it is rarely defined anywhere. It is difficult to define mental illness unless mental health is first defined. Based upon the first author and the United States Surgeon General’s Mental Health Report, mental health is present when mental functioning facilitates adaptive and productive activities with purpose and meaning, fulfilling relationships and the capacity to enjoy the activities of life, the capacity to contend with adversity, and the mental flexibility to adapt to changing life circumstances [64,65]. A systems approach expands upon the biopsychosocial model used in psychiatry and helps to organize the multiple systems that contribute to human functioning in both health and disease [64]. Some of these systems have been categorized with the suffix “ome”, such as genome, proteome, microbiome, infectome, metabolome, etc. The study of these respective fields uses the “omics” suffix. For example, genomics, proteomics, etc. [66]. 

#### 3.2.2. Mental Illness, Mental Disorder

Mental illness is also called psychiatric illness and mental disorder. The APA DSM-5 defines a mental disorder as “a syndrome characterized by clinically significant disturbance in an individual’s cognition, emotion regulation, or behavior that reflects a dysfunction in the psychological, biological, or developmental processes underlying mental functioning” [54]. The ICD defines a mental disorder as “a clinically recognizable set of symptoms or behaviours associated in most cases with distress and with interference with personal functions” [55]. Another definition by the first author and based upon the United States Surgeon General’s Mental Health Report defines mental illness as an impairment of adaptive capabilities that impedes productive activities, purpose and meaning, fulfillment of relationships, and the capacity to enjoy the activities of life, the capacity to contend with adversity, and the mental flexibility to adapt to changing life circumstances [64,65]. 

The APA DSM-5 categorizes—but does not address—the causes of mental illnesses. Mental illness is the result of an interaction of multiple contributors and susceptibilities resulting in a pathophysiological process. This can result in a combination of cognitive (cortical), emotional (limbic), and/or vegetative (brain stem) impairments. Using a systems model, mental functioning can be conceptualized as being a balance of multiple contributors and deterrents that result in either mental health or mental illness. Mental illness is associated with an imbalance between these contributors and deterrents with a net effect that leads to a sequential pathological process. The time sequence may consist of an interaction of predisposing and precipitating contributors resulting in immune, neurochemical, and/or other changes that cause a pathophysiological process. That process may result in dysfunction that may cause mental symptoms and syndromes. Diagnostic and treatment delays can result in a perpetuation of disease progression and an increase in disease severity. This process results in the disorders listed in the APA DSM-5 [64]. 

Many of the mental disorders listed with different codes and defined in the APA DSM-5 can be conceptualized as dysregulated and excessive, aversive emotional states [67]. From this perspective, these dysregulated, aversive emotional states include environmental phobias (agoraphobia, claustrophobia, acrophobia, etc.); interpersonal (paranoia, social anxiety, body dysmorphic disorder, pathological jealousy, etc.); body integrity (somatic symptom disorder, illness anxiety disorder); traumatic reactivity (posttraumatic stress disorder); alarm (panic disorder); doubt (obsessive compulsive disorder); grooming disorders (trichotillomania, excoriation disorder, onychophagia, rhinotillexomania, body cleaning compulsiveness); and depression (futility) [67].

#### 3.2.3. Psychosomatic Disorders

The term psychosomatic is not in the APA DSM-5 [51]. The DSM-5 only classifies symptoms and syndromes but does not address causality. As a result, psychosomatic disorders are not identified, defined, or explained in the DSM-5 [54]. The ICD addresses causality with some diagnostic categories but does not address psychosomatic causality [55]. Since the APA DSM-5 does not address psychosomatic conditions and the ICD only partially addresses psychosomatic conditions, there are gaps in standardizing the definition and the classification of psychosomatic disorders. 

Psychosomatic disorders are somatic illness caused or exacerbated by mental stress and distress. The list of conditions considered to have a purely psychosomatic basis keeps shrinking as scientific knowledge advances. Tuberculosis, hypertension, and stomach ulcers were all once considered as having a psychosomatic etiology. However, it is recognized that many diseases have psychosomatic contributors and are made worse by stress and distress, such as heart disease, irritable bowel syndrome, nervous stomach, and skeletal muscle guarding [67].

When stress occurs in an individual who is more emotionally and physiologically reactive, there will be an increased allostatic load (wear and tear on the body from stress) with accompanying physiological changes. These changes may include: (1) a shift in the autonomic nervous system balance from parasympathetic to sympathetic control; (2) changes in the hypothalamic-pituitary-adrenal axis; (3) increased blood pressure, heart rate, breathing; (4) increased blood glucose; (5) increased blood flow to skeletal muscles; (6) inflammation; (7) decreased regenerative (recovery) activity; (8) decreased digestive activity; and (9) decreased blood flow to the prefrontal cortex at higher levels of distress [68,69,70]. Although brief episodes of acute stress can generally be healthy and well tolerated in most, chronic unremitting stress in susceptible individuals can have a more deleterious effect. In an individual with genetic and other susceptibilities to stress, these changes may in turn result in psychosomatic symptoms and disorders. Individuals have different vulnerabilities that make them more prone to different psychosomatic conditions.

One example of a psychosomatic illness is psychosomatic cardiovascular disease. When this occurs in a susceptible individual, chronic stress activates the hypothalamic–pituitary–adrenal axis and the sympathetic branch of the autonomic nervous system, reduces vagal tone, increases plasma catecholamines, elevates heart rate, causes vasoconstriction, activates platelets, and reduces heart rate variability [71]. Associated chronic increases in proinflammatory cytokines contribute to endothelial damage, plaque formation, atherosclerotic thrombus formation, vascular occlusion, endothelial damage of the cerebral vasculature, and acute coronary syndromes. These autonomic and immune system changes singly and additively exert adverse effects, resulting in high cardiovascular morbidity and mortality [71].

Another example is irritable bowel syndrome. In a susceptible individual, stress results in reduced parasympathetic and vagal tone, and peristaltic contractions become more spastic, resulting in diarrhea, bowel urgency, and/or constipation. Dietary considerations, particularly gluten and lactose, also play a role in symptom exacerbation [72,73].

#### 3.2.4. Somatopsychic Disorders

The term somatopsychic is not in the APA DSM-5 or the ICD. As a result, somatopsychic disorders are not defined and explained by formally used diagnostic systems, which leaves a gap in standardizing the definitions and the classification of somatopsychic disorders. Somatopsychic disorders are mental disorders caused or exacerbated by somatic disorders. In contrast to psychosomatic disorders, the list of somatic conditions causing mental disorders keeps expanding as scientific knowledge advances. Many general medical conditions are recognized as causing psychiatric symptoms. Endocrine disorders, tumors, autoimmune disorders, and infections are particularly associated with causing psychiatric symptoms. Thousands of peer-reviewed journal articles demonstrate the causal association between infections, somatic illness, and mental illness. Most of these symptoms are immune mediated. The identified infectious triggers include viral, venereal, and vector-borne diseases [74,75]. 

#### 3.2.5. Multisystem Disorders

Multisystem disorders are not addressed in the APA DSM-5. Multisystem disorders are conditions that impact the entire body and cause symptoms in multiple systems, such as the nervous system, the immune system, the endocrine system, etc. In these conditions, there are both somatic and psychiatric symptoms. Sometimes, there is not a clear distinction between a somatopsychic and a multisystem disorder. The list of multisystem disorders associated with mental disorders keeps expanding, while the list of purely psychosomatic illnesses keeps shrinking as scientific knowledge advances. Multisystem disorders include deficiencies, toxic states, systemic infections, and systemic immune disorders. Schizophrenia, bipolar disorders, and other mental disorders are not exclusively neuropsychiatric disorders. There is mounting evidence that these are multisystem disorders with immune-mediated metabolic components as well [76,77,78,79].

#### 3.2.6. Medical Uncertainty

Medical uncertainty is defined as a “subjective perception of an inability to provide an accurate explanation of the patient’s health problem” [80]. Medical uncertainty is not a diagnosis. Instead, there is always some degree of certainty or uncertainty with any medical condition or diagnosis. There is still much to learn about illness and the brain–body interaction. No medical condition is totally explained or unexplained. Instead, knowledge is on a continuum, and all conditions are partially explained to different degrees [54]. As a result, some degree of uncertainty always has been, and always will be, a part of medicine. Diseases that are relatively easier to understand present with simpler and more clearly defined causes, pathophysiology, and symptoms. The more challenging diseases are those that are more complex and are often considered more controversial. They have multiple disease contributors, pathophysiology, and symptom presentations with a greater amount of medical uncertainty.

The many contributing factors to medical uncertainty include the uniqueness of individuals, biological heterogeneity, and the complexity of conditions. These result in variability of disease presentations and individual differences in treatment effectiveness, tolerability, and safety. Donald Rumsfeld summarized the uncertainty dilemma by stating, “There are known knowns; there are things we know we know. We also know there are known unknowns; that is to say we know there are some things we do not know. But there are also unknown unknowns—the ones we don’t know we don’t know… it is the latter category that tends to be the difficult one.” [81]. Medical uncertainty may result in patient and physician bias, error in test interpretation, differing values and opinions between patients and physicians, and uncertainty surrounding decision-making.

#### 3.2.7. Diagnostic Terms

##### Relevant Mental Disorders Recognized by the American Psychiatric Association Diagnostic and Statistical Manual

The American Psychiatric Association DSM-5 is a well-recognized source for definitions of mental disorders. There are several mental disorders that are particularly relevant to diagnostic controversies regarding the brain–body interface. These conditions include somatic symptom disorders, somatoform disorders, functional neurological symptom disorder, illness anxiety disorder, factitious disorder, and factitious disorder imposed upon another. In addition, anxiety disorders, obsessive-compulsive disorders, trauma-related disorders, and stressor-related disorders are frequently associated with psychosomatic symptoms. The diagnostic categories that are being discussed shall be followed by the APA DSM-5 code and the ICD code in parentheses. The diagnostic categories in the ICD 10th and the 11th Revision in most cases, closely follow the APA DSM-5 in defining mental disorders [54,55]. The one major distinction is the inclusion of bodily distress disorder in the proposed 11th Revision [55].

##### Somatic Symptom Disorders

Somatic symptom disorders are included in the APA DSM-5 and are associated with excessive thoughts, feelings or behaviors related to somatic symptoms and one of three of the following criteria which need to be present for at least six months: (1) health anxiety, (2) disproportionate and persistent concerns about the medical seriousness of the symptoms, and (3) excessive time and energy devoted to symptoms or health concerns [54]. Somatic symptom disorders include somatic symptom disorder, 300.82 (F45.1); illness anxiety disorder, 300.7 (F45.21); functional neurological symptom disorder, 300.11 (F44); factitious disorder, 300.19; (F68.10); psychological factors affecting other medical conditions, 316 (F54); other specified somatic symptoms and related disorders, 300.89 (F45.8) and unspecified and related disorders, 300.82 (F45.9). Unlike somatoform disorders, the physical symptoms may or may not be associated with a diagnosed medical condition [54]. The APA DSM committee considered the prior term, somatoform disorder, in the APA Diagnostic and Statistical Manual, Fourth Edition, Text Revision (DSM-IV-TR). It was inaccurate and outdated since it was dependent upon “medically unexplained symptoms.” [54,82]. The term “somatic symptom disorders” improves upon and replaces somatoform disorder. In the APA DSM-5, a person is not diagnosed with somatic symptom disorder solely because a medical cause can’t be identified for a physical symptom. The basis of the diagnosis is instead dependent upon the extent to which the thoughts, feelings and behaviors related to the illness are excessive or out of proportion as subjectively determined by the evaluating physician [54].

##### Somatoform Disorders and Medically Unexplained Symptoms

Somatoform Disorders

Somatoform disorders were once considered to be a psychiatric condition marked by multiple, medically unexplained, physical, or somatic symptoms. The category of somatoform disorders and the diagnosis of somatization disorder were listed in the APA DSM-IV-TR [82]. The term “somatoform” was used when there was a belief that the physical symptoms had a psychological origin. Both terms were removed when the DSM-IV-TR was updated to the DSM-5. To have met the diagnostic criteria for somatization disorder, somatic complaints must have also been serious enough to interfere significantly with a person’s ability to perform important activities, such as work, school, or family and social responsibilities, or lead the person experiencing the symptoms to seek medical treatment [82].

Medically Unexplained Symptoms

“Medically unexplained symptoms” is a term that is no longer valid in the APA DSM-5 [54]. They were physical symptoms for which a treating healthcare provider had found no medical cause or where the cause remained contested, unknown, or disputed. Most who used the term considered the symptoms had to be of a psychological origin. The phrase “medically unexplained symptoms” never was a diagnosis but was used in the DSM-IV-TR to refer to the criteria used to diagnose somatoform disorder. It is now outdated and is not included as being relevant in the diagnosis of somatic symptom disorder in the APA DSM-5. An explanation for eliminating this phrase is the recognition that no medical condition is totally explained or unexplained; instead, knowledge is on a continuum, and all conditions are partially explained to different degrees. This label is impacted by the bias and the level of knowledge of anyone calling it “unexplained”. These symptoms are often unexamined rather than unexplained [54].

##### Functional Neurological Symptom Disorder

Functional neurological symptom disorder, APA DSM-5 300.11 (F44), involves one or more symptoms of altered voluntary motor or sensory function [54]. It was previously called conversion disorder. An example would be the paralysis of an arm after striking a family member during an argument. The psychodynamic explanation is unconscious repression of intrapsychic conflicts resulting in a conversion into a physiological symptom, such as hysterical blindness or paralysis. Although the diagnosis of conversion disorder is given freely by some physicians, actual cases are only rarely seen in developed countries [83].

##### Illness Anxiety Disorder

Illness anxiety disorder, APA DSM-5 300.7 (F45.21), previously called hypochondriasis, is a preoccupation or excessive concern with acquiring a serious illness [54]. An example would be a fear of acquiring acquired immunodeficiency syndrome (AIDS) from using a public swimming pool. This contrasts with somatic symptom disorder in which there is an excessive concern regarding symptoms that currently exist. Cyberchondria is not an APA DSM diagnosis but may be a category of illness anxiety disorder that occurs when there is excessive illness anxiety associated with using the Internet for healthcare information [84]. The Internet has made medical information more available to the public, and individuals with limited medical knowledge sometimes have difficulty interpreting medical information in the proper context. Illness anxiety disorder can sometimes be confused with normal health concerns.

##### Factitious Disorders, Factitious Disorder Imposed on Another

Factitious disorders, APA DSM-5 300.9 (F68.10), includes factitious disorder imposed on the self, which is also called Munchausen’s syndrome, consists of the falsification of physical or psychological signs or symptoms or the induction of injury or disease associated with identified deception upon oneself. For example, a person injects a foreign substance into themselves to contrive an illness that would not otherwise exist. 

Factitious disorder imposed on another (also called Munchausen’s by proxy), APA DSM-5 300.9 (F68.10), is the intentional production of symptoms in another person that consists of the falsification of physical or psychological signs or symptoms or the induction of injury or disease associated with identified deception. For example, a parent injects a foreign substance into their child to contrive an illness that would not otherwise exist. True cases of factitious disorder and factitious disorder imposed on another are extremely rare. The DSM-5 criteria require contrived deception, not disagreement about the diagnosis or the seriousness of the symptoms [54].

Factitious disorder imposed on another is a highly controversial diagnosis. Most allegations involve a single parent—but sometimes both parents—and it may involve one or more children. In these cases, disagreement between a parent or parents and the treating pediatrician sometimes result in an improper diagnosis of factitious disorder imposed on another. False allegations of factitious disorder imposed on another usually involve a child with an orphan disease or a complex disease not adequately understood by the physician giving the diagnosis. Sherr described this problem by stating, “Physicians unfamiliar with Lyme patients’ shifting, seemingly vague, emotional, and/or bizarre-sounding complaints, frequently know little about late-stage spirochetal disease” [85]; she continued, “Consequently, they may accuse mothers of fabricating their children’s symptoms—the so-called Munchausen’s by proxy ‘diagnoses’…Many such cases involve an unrecognized Lyme borreliosis causation that mothers may insist is valid despite negative tests”. Many children sick from complex diseases have been forcibly removed from their parents who insist, contrary to the pediatrician’s evaluation, that their children are ill. The charges against these parents accuse them of believing their children are sick because of their own psychopathology [85].

In some countries such as the Netherlands, children with chronic or complex illnesses such as Lyme disease are sometimes removed from their parents by Child Protection Services. Officials believe that these children are victims of Munchausen’s Syndrome By Proxy. Out of concern for the increase in false accusations of Munchausen By Proxy, an advocacy group for chronically ill children has documented over 300 such cases in the Netherlands. In around one-third of these cases, the child had Lyme disease. These cases have been recorded as human rights violations by the United Nations [86]. Factitious disorder upon another is a contrived illness. It is not a disagreement about a diagnosis or the seriousness of an illness. It is possible to contrive a tumor by injecting a foreign substance, but it is difficult to imagine how anyone could contrive the multiple symptoms associated with Lyme disease or other complex multisystem illnesses.

#### 3.2.8. Functional Disorders

Functional disorders have never been included in any edition of the American Psychiatric Association Diagnostic and Statistical Manual. However, functional intestinal disorder, unspecified, K59.9, and unspecified functional disorder of stomach, 536.9, were included in the ICD-10. A functional disorder is viewed as a medical condition that impairs normal functioning of bodily processes and remains largely undetected under physical examination, dissection, or by microscopic examination. To meet the definition, there must be no exterior appearance of abnormality. A functional disorder contrasts with a structural disorder in which some part of the body is seen as being abnormal. The mechanism that causes a “functional disorder” is generally considered to be unknown or poorly understood. Examples include chest pain, fatigue, pain, and/or many other symptoms that cannot be confirmed with confirmatory testing within the clinical capabilities of the examining physician. An inadequate assessment can result in an inaccurate diagnosis of a functional disorder.

#### 3.2.9. Psychogenic Disorders

Psychogenic disorders were never included in any edition of the American Psychiatric Association Diagnostic and Statistical Manual. However, the term is included in ICD-10 as other somatoform disorders, F45.8 [87]. Based upon ICD-10, conditions that can be categorized as being psychogenic include aerophagy, bruxism, cardiovascular disorder, constipation, cough, dissociative convulsions, dysmenorrhea, dysuria, gastrointestinal malfunction, genitourinary malfunction, globus hystericus, globus sensation, hyperventilation, musculoskeletal disorder, neurocirculatory asthenia, pruritus, pseudocyesis, seizures, teeth grinding, torticollis, and vocal cord dysfunction [87].

Psychogenic disorders are physical illnesses that are believed to have been caused by emotional or mental stressors or consequences of psychiatric or psychological disorders. In addition, it can be a physical abnormality or other biomarker that cannot be identified or cannot be explained by confirmatory testing within the capabilities of the examining physician. 

It is a valid concept that physical illnesses can be caused or exacerbated by emotional or mental stressors by psychiatric or psychological disorders. However, the criterion that there is no physical abnormality or other biomarker that can be identified or explained is becoming an increasingly less valid concept. Since the development and the expansion of brain imaging, neurochemistry, microarray technology, improved testing for somatic illnesses, and other advances, pathological changes can now be better identified. These pathological changes can be more readily identified in general medical as well as psychiatric illnesses. The absence of pathological anatomical findings may sometimes strengthen the possibility of a psychiatric or a psychosomatic illness, but the absence of a finding alone can never confirm the presence of a psychiatric illness. A more thorough psychiatric assessment can determine whether there is a psychodynamic and/or psychiatric pathophysiological process that can explain the etiology of a symptom. No diagnosis is a diagnosis by default, including the diagnosis of mental illness. Despite this, it is a common practice to label a poorly understood condition as being psychogenic, even when there may have been no psychiatric evaluation, or an inadequate psychiatric examination with no evidence of a psychiatric etiology, or a competent psychiatric evaluation that reveals no evidence of psychiatric illness.

#### 3.2.10. Compensation Neurosis

“Compensation neurosis”, also called by numerous synonyms, has never been included in any edition of the American Psychiatric Association Diagnostic and Statistical Manual. It is not included in ICD. It is considered a neurosis associated with wanting compensation from an insurance company. Neurosis is an outdated term that was defined as a relatively mild mental illness that is not caused by organic disease and involves symptoms of stress that may be depression, anxiety, obsessiveness, or hypochondriasis but without a radical loss of touch with reality. The term “compensation neurosis” has been used for many years by experts paid by insurance companies when discussing the emotional sequelae of accidental injury victims [88]. The clinical validity of this term is without any scientific support for diagnosis and classification, and there are ethical questions in the literature regarding the use of this term as a diagnosis. All examinations of "compensation neurosis" as an illness entity, using standard criteria of diagnostic validity, do not support the view that such a distinct disease exists [88].

#### 3.2.11. Psychogenic Seizures

Psychogenic seizures were never included in any edition of the American Psychiatric Association’s *Diagnostic and Statistical Manual of Mental Disorders*. However, the term is listed in ICD. Psychogenic seizures are also called psychogenic nonepileptic attacks, psychogenic nonepileptic seizures, dissociative seizures, and pseudoseizures. During these episodes, patients manifest complex partial seizure activity, but seizure activity is not demonstrated on electroencephalograms (EEG). It is therefore, considered a diagnosis by exclusion [89]. These episodes may be accompanied by myoclonic jerks. No evidence can be found demonstrating a psychodynamic explanation for these seizures or the myoclonic jerks that may accompany these episodes.

Physicians have mixed opinions regarding the etiology of what is called psychogenic seizures [90]. Unfortunately, partial seizure activity that is localized deep within the brain cannot always be measured with the current diagnostic technology that measures seizure activity on the surface of the brain [91]. A thorough history and clinical assessment, nasopharyngeal leads, sleep EEG recordings, 24-hour EEG monitoring, computerized EEGs, single-photon emission computed tomography, video-electroencephalogram, and empirical treatment with anticonvulsants can result in a diagnosis of complex partial seizures in many who were previously diagnosed as having psychogenic seizures. Emotional distress and hyperventilation can lower seizure threshold in a patient who is prone to seizure activity. However, when emotional distress lowers seizure threshold, it is a psychiatric contributor but not a true psychogenic seizure. 

#### 3.2.12. Psychogenic Pain

Psychogenic pain is not an APA DSM diagnosis. It is considered to be a pain disorder that is associated with psychological factors. A patient who is given this diagnosis is viewed as having complaints of pain that do not match the symptoms recognized by the evaluating physician. It is considered that some mental conditions, such as anxiety and depression, may increase the focus upon and the sensitivity to pain. It is a diagnosis by default and is made only when all other causes of pain have been ruled out [92]. Pain and fatigue, which are considered to have a psychiatric origin, are believed to be significant symptoms in diagnosing bodily distress disorder.

Fear, anxiety, and depression can clearly exacerbate a perception of pain [93,94,95]. Emotional distress can result in muscle guarding and autonomic reactions that cause pain [96,97]. In these cases, it is important to clarify the psychodynamics and the pathophysiological processes that result in the perception of pain. However, it is important not to confuse psychogenic pain with undiagnosed medical conditions and radiculopathy, neuropathy, neuropathic pain, hyperalgesia and allodynia, all of which have a neurological and/or immune basis [98,99,100,101,102].

#### 3.2.13. Delusional Parasitosis

Delusional parasitosis or delusions of parasitosis is not an APA DSM-5 diagnosis. It is a rigidly fixed belief of being infested with pathogens, even when presented with evidence and appropriate reassurance to the contrary. It is not a single disorder but can be secondary to numerous other conditions [103]. The closest DSM-5 diagnosis is delusional disorder, 297.1 [54]. However, most patients with delusional disorder have multiple delusions, not a single delusion such as parasitosis. The closest ICD-10 diagnosis may be psychotic disorder with delusions due to a known physiological condition (F06.2) [104]. 

Some patients complain of formication, which is a sensation of crawling under the skin. Recognized somatic causes of formication include menopause, pesticide exposure, reactions to dental chemicals, mercury poisoning, diabetic neuropathy, skin cancer, syphilis, Lyme disease, Morgellons disease, herpes zoster (shingles), alcohol withdrawal, and stimulant intoxication with methamphetamines or cocaine [105,106]. Although patients experiencing formication describe it as a stinging sensation with a sensation of bugs crawling under their skin, most patients with formication can be reassured that the sensation of bugs under their skin is instead a neurological symptom. Delusional parasitosis can easily be confused with Morgellons disease, which is a skin condition characterized by the presence of multicolored filaments that lie under, are embedded in, or project from skin. Clinical studies supporting the opinion that Morgellons disease has a delusional etiology have considerable methodological flaws and often neglect the fact that mental disorders can result from underlying somatic illness. By contrast, rigorous experimental investigations have shown that this skin affliction results from a physiological response to the presence of an infectious agent. Investigations have determined that the cutaneous filaments found in these patients are composed of the cellular proteins, keratin and collagen, and result from overproduction of these filaments in response to spirochetal infection [107,108].

#### 3.2.14. Subjective vs. Objective Complaints and Symptoms

Symptoms such as fatigue, aches, pain, cognitive impairments, mood dysregulation sensory complaints, etc., are categorized by some as “subjective” and argued by some to be less valid [40,109]. Conversely, laboratory tests, even when poorly standardized (such as two-tier Lyme disease testing), and clinical trials, even when poorly designed (such as the Klempner Lyme disease study), are categorized by some as “objective” and, therefore, considered by some to be more valid [40,41,42,43,44,45,46,47,48,110,111,112]. This belief system was quite evident in both the IDSA Lyme disease guidelines and the review of the guidelines [40,109]. 

#### 3.2.15. Non-Specific and Vague Symptoms

Complex diseases can have different presentations in different individuals with symptoms that may overlap with other conditions. Many of these symptoms may by themselves not be specific to a unique diagnosis. The term “vague” is also sometimes used to categorize symptoms that are not diagnostically specific but can be very serious symptoms. These symptoms may include unexplained weight loss and/or appetite loss, non-specific abdominal discomfort, or pain, fatigue, cognitive impairments, sensory complaints, mood dysregulation, and excessive sweating [113]. 

Some healthcare providers have great difficulty understanding and making an accurate diagnosis when these symptoms are present and categorize them as being “vague” or “subjective” symptoms and, therefore, less valid. Conversely, laboratory tests, even when poorly standardized, and clinical trials, even when poorly designed, are categorized by some as “objective” and are, therefore, argued to be more valid. Fatigue, pain, cognitive impairments, mood dysregulation, and sensory impairments are complaints that can be validated objectively by a competent clinician and can be confirmed with mental status evaluations, psychological testing, several measurement scales, and brain imaging [114,115,116]. Vague and non-specific symptoms can often indicate immune activation in response to chronic infections, cancer, and other serious conditions [57,59,117,118].

Complex diseases can have different presentations in different individuals with symptoms that may overlap with other conditions, and many of the complex disease symptoms may by themselves not be significantly specific for a unique diagnosis. Although many symptoms may superficially be viewed as vague and non-specific, it is possible to recognize patterns of these symptoms accompanied by disease progression specific to a condition.

#### 3.2.16. Bodily Distress Disorder, Bodily Distress Syndrome

Bodily distress disorder is closely related to bodily distress syndrome. Neither are included in any edition of the APA DSM or in the ICD-10. The medically unexplained symptoms criteria for somatoform disorder have been criticized for being unreliable, since they define a disorder based on the absence of identifying features rather than the recognition of a problem [119]. In the transition from somatoform disorder to somatic symptom disorder, the most significant change was the removal of the invalid distinction between medically explained and medically unexplained somatic complaints. A group of proponents in Europe salvaged the diagnostic category with a substitute phrase and were able to have it listed in the proposed ICD-11 (6C20). These proponents renamed it bodily distress disorder and replaced the medically unexplained criteria with the concept of long-standing excessive distress and excessive thoughts, and behaviors towards pain that are considered of either known or unknown etiology [120]. In contrast, bodily distress syndrome is associated with excessive thoughts and behavior that are considered of unknown (medically unexplained) etiology [120,121].

The current ICD-11 draft, dated April 2019, states, “Bodily distress disorder involves bodily symptoms that the individual finds distressing and to which excessive attention is directed” [122]. In literature advocating for this diagnosis, this distress can lead to mutual distress on the part of both the patient and the doctor, “as well as costing a lot of money for the healthcare system” [119]. This excessive attention is not alleviated by “appropriate clinical examination or investigations and appropriate reassurance”. Bodily symptoms are also persistent, being present on most days for at least several months. Typically, bodily distress disorder involves multiple bodily symptoms that may vary over time. Occasionally, there may be a single symptom, such as pain or fatigue. Patients with bodily distress disorder are seen as having medically unexplained or functional symptoms and include a range of what these proponents consider to be poorly defined disorders, including chronic fatigue syndrome (ME/CFS), fibromyalgia, irritable bowel syndrome, chronic pain syndrome, hyperventilation syndrome, non-cardiac chest pain, and somatoform disorder [119,120,123,124]. 

In the definition of bodily distress syndrome, there is a group of conditions that have little in common other than being distressing to deal with by some physicians. This group includes chronic fatigue syndrome (ME/CFS), fibromyalgia, irritable bowel syndrome, chronic pain syndrome, hyperventilation syndrome, non-cardiac chest pain, and somatoform disorder. What these conditions are considered to have in common is the belief that there is a central sensitization syndrome, which is not supported by any neurophysiological evidence. In addition to this deficiency, there is a failure to be scientifically defined as a diagnostic category, and from an evidence-based medicine perspective, it fails to establish that it excludes patients with medical conditions that require medical care. The flaws in the concept of bodily distress disorder and bodily distress syndrome are like the flaws that were revealed in the PACE study: Labeling patients in this manner results in poor treatment outcomes [125,126]. Allen Frances, Chair of the DSM-IV Task Force, stated that bodily distress disorder in the ICD-11 is a "bad mistake because it: (1) will mislabel as mentally ill millions who have normal health worry; (2) allows docs to assume ‘It’s all in your head’; (3) encourages inadequate medical testing/diagnosis; (4) weak research; (5) wide patient opposition; 6) repeats DSM error” [127].

Since neither bodily distress disorder nor bodily distress syndrome are included in the DSM-5, and there is no indication it will ever be recognized as a valid diagnosis by the American Psychiatric Association, it poses a somewhat lesser threat to patients in the United States. Its inclusion in ICD-11, however, can particularly impact other countries. There may be a motivation to label patients with bodily distress disorder with a belief it will reduce short-term healthcare costs. Instead, it may have a long-term adverse effect upon the health of tens of millions of suffering patients across the globe, which makes it a concern for ethics as well as science [125].

## 4. Discussion

### 4.1. Significant Issues

If the sequential cause of a patient’s illness is better identified and understood, it improves the opportunity for more effective diagnosis, treatment, and healthcare outcomes by healthcare providers. In addition, better insight in this area may help prevent errors and inaccuracies on the part of third parties, which may otherwise lead to misdirected financial resources and regulatory effort. 

A review of the definitions raises some significant issues that need further discussion. This includes cause/effect vs. interactive relationships, multisystem vs. psychosomatic disorders, “excessive” concern regarding symptoms, whether fatigue can be psychogenic, ethical concerns, and adequacy of assessment. Many of the terms discussed are shown in Table 1 and have the potential to be misused and abused.

### 4.2. Cause, Effect vs. Interactive Relationship

**Figure 1:**
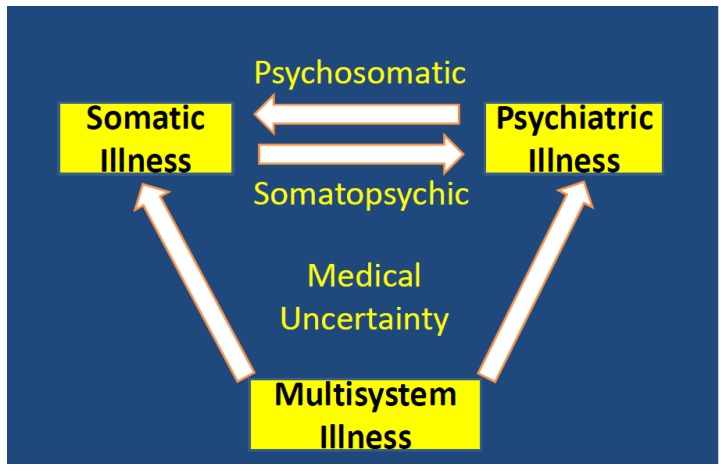
The relationship between psychosomatic, somatopsychic, multi system illness, and medical uncertainty.

In any given situation, it is difficult to determine if emotional distress causes somatic symptoms, somatic distress causes emotional symptoms, or a multisystem condition causes both. There may also be a very complex cause–effect relationship, or there may be a high level of true medical uncertainty regarding the cause and effect relationship. The medical uncertainty may be impacted by the limited knowledge of the examining doctor. This is demonstrated in Figure 1. 

### 4.3. Multisystem vs. Psychosomatic Disorders

As was demonstrated in the two case histories, a person can be reasonably healthy previously or throughout most of his/her life, and then a multitude of symptoms progressively appear that subsequently expand in number and severity. The number and the complexity of these symptoms may be overwhelming to the patient, and the patient may be labeled as being hypochondriacal, having a psychosomatic illness, or having bodily distress disorder or somatic symptom disorder. However, more commonly, hypochondriasis and psychosomatic illnesses begin in childhood and are lifelong conditions that vary in intensity depending upon life stressors [128,129,130]. If a complex illness with a multitude of both mental and physical components begins later in life, the likelihood that this is an immune mediated, multisystem disorder is greater than it being a psychosomatic disorder [131,132].

### 4.4. Excessive Concern Regarding Symptoms

“Excessive” is the critical word in the bodily distress diagnosis. The name bodily distress disorder implies there is distress associated with bodily functioning to such an excessive degree that it is called a disorder. “Excessive” is also used in the diagnosis of somatic symptom disorder. “Excessive thoughts, feelings, or behavior related to somatic symptoms, which have been present for at least six months” is a criterion for somatic symptom disorder in DSM-5 [54]: “Bodily symptoms that are distressing to the individual and excessive attention directed toward the symptoms…not alleviated by appropriate clinical examination and investigations and appropriate reassurance…persistent…at least several months…usually pain or fatigue”. There are proposed criteria for bodily distress disorder in ICD-11 [55]. No objective criteria exist to identify how “excessive” concern is defined. The definition can easily be impacted by the limits of the examination, the conceptual abilities, the bias of the examining physician, or the financial goals of the insurance company or the single payer. Some patients with Lyme disease are viewed as having an excessive concern for their symptoms. The IDSA Lyme disease guidelines dismissed the chronic complaints from Lyme disease as being the “aches and pains of daily living” [40]. In contrast, using objective criteria, a National Institute of Health study found chronic Lyme disease patients had pain comparable to post-surgical pain, and fatigue comparable to multiple sclerosis patients [133]. If a previously healthy and active person acquires a debilitating, multisystem condition, with multiple complaints including paralyzing fatigue and pain that adversely impact multiple areas of functioning, and the evaluating physician has an inadequate knowledge of the illness, takes an inadequate history, performs an inadequate exam, and does not understand the seriousness of symptoms, or fails to use adequate clinical judgment, how can the patient’s response to the physician’s “reassurance” be considered excessive?

### 4.5. Can Fatigue Be Psychogenic?

Fatigue is a lack of energy unrestored by rest [134,135]. It is the second most common presenting medical complaint in a primary care physician’s office after chest pain. Fatigue can commonly be associated with a proinflammatory state and sickness syndrome, which can be evoked by infections, cancer, allergies, injury, etc. There is no evidence that fatigue associated with myalgic encephalomyelitis/chronic fatigue syndrome (ME/CFS), Lyme disease, or other chronic multi-system illnesses is psychogenic. In contrast, fatigue can cause mental distress. In the progression of multisystem illness, fatigue is an earlier symptom, and depression is usually a later and less common symptom. Fatigue as well as sickness syndrome (without fever and chills) can be a part of major depression [54]. Although fatigue is associated with major depression, it is difficult to find a psychodynamic explanation that fatigue can have a psychogenic basis.

### 4.6. Ethical Concerns

Maintaining respect for the patient, protecting the integrity of the physician–patient relationship, and preserving individualized healthcare are high priorities in healthcare. Issues that have the potential to undermine the adequacy of assessment and medical judgment include third party intrusions with pressure for adherence to dogmatic, third-party controlled diagnostic and treatment guidelines, an incorrect application of research findings to individualized situations, flawed guidelines, and economic pressures that limit the adequacy of assessment. Guidelines based upon inaccurate terms and concepts harm patients, especially when they are accompanied by efforts to convert the guidelines into standards of care [40,136]. Failures in these areas can result in misdiagnosing someone who has a multisystem disorder with an erroneous diagnosis of somatic symptom disorder or some other error [137].

Bodily distress syndrome and bodily distress disorder are ethically distressing concepts. How does one defend the persistent investment in labeling patients with medically unexplained symptoms and/or bodily distress disorder? There is an ethical mandate based upon the Hippocratic Oath to defer to the needs of the patient when conflicts of interest arise. Some nomenclature in flawed diagnostic categories appears to be designed to protect the best interests of third parties and/or the treating physician’s distress rather than the best interest of the patient by denying the validity of highly significant symptoms and a more thorough assessment and treatment of the patient’s complaints. This raises ethical concerns with how we make diagnoses: When making a diagnosis of bodily distress disorder, whose distress are we really trying to relieve? If the patient complains about a poor outcome, does this diagnosis relieve the physician’s distress from following a flawed belief system or the distress when conflicting financial interests are jeopardized? Is the physician’s distress then protected by labeling the patient’s complaints as subjective and non-specific, “the aches and pains of daily living”, medically unexplained symptoms, excessive concern, excessive body distress, and/or bodily distress syndrome? A more valid term might be diagnostic distress syndrome. In science and medicine, when a finding is incompatible with a hypothesis and diagnosis, the hypothesis and diagnosis need to be questioned. 

### 4.7. Adequacy of Assessment

Clarifying the mind–body interaction in any given patient requires adequate psychiatric and general medical assessments. The overall clinical exam is the cornerstone of medicine and is the foundation for every patient’s individualized treatment. This approach has been a concept in medicine that has existed since Hippocrates. Sir William Osler, who is considered the Father of American Medicine, emphasized the significance of clinical observation, a thorough examination and individualized judgment when assessing a patient. Some of the better-known aphorisms supporting this position include [138]:

“There is no more difficult art to acquire than the art of observation.”

“If you listen long enough, the patient will give you the diagnosis.”

“Medicine is learned by the bedside and not in the classroom. Let not your conceptions of disease come from the words heard in the lecture room or read from the book. See and then reason and compare and control. But see first.”

“To study the phenomena of disease without books is to sail an uncharted sea, while to study books without patients is not to go to sea at all.”

“The greater the ignorance, the greater the dogmatism.”

The Institute of Medicine has formalized a similar position by defining evidence-based practice as “the integration of best-researched evidence and clinical expertise with patient values” [139]. 

In medicine, we treat patients, not diseases. Patient care is compromised when medical practices emphasize population-based standards of care rather than individual patient needs and experiences [140]. It is a concern that current clinical practice guidelines, which many doctors follow, are aimed primarily at managing single diseases. These guidelines are of little help in aiding physicians when it comes to treating patients who have multiple conditions. In addition, many of the clinical guidelines are written by disease-specific specialists who may not consider the clinical picture beyond their area of specialization. Because of these issues, physicians should rely less on clinical guidelines for managing single diseases and more on their own clinical judgment to create treatment plans that meet the needs of individual patients [5]. Caring and empathy are also critical components of the physician–patient relationship. These are sometimes overlooked when there is an excessive emphasis upon the scientific component of the practice of medicine [141].

The best researched scientific evidence available can be contradictory or equivocal. Scientific research is perhaps viewed as being on a continuum of different degrees of knowledge. When new evidence occurs, it can challenge the legitimacy of previous beliefs and scientific hypotheses. Diagnostic and treatment guidelines of different degrees of reliability are sometimes established to offer some assistance in clinical decision making. However, all guidelines should have a clear disclaimer stating that guidelines are not a replacement for prudent clinical judgment. The Great Britain National Health Service views itself as having a strong scientific tradition of evidence-based decisions about care: “But tradition should be a foundation for growth, not a straitjacket. A forward-looking National Health Service would recognize that patient experience evidence should be respected, cherished, and used on an equal footing with medical evidence. It is time for the double standard to end.” [142].

## 5. Conclusions

Historically, there has been a tendency to label physical symptoms that could not be explained as being of a psychiatric origin. As a result, many patients with complex, confusing symptoms and poorly understood diseases who receive an inadequate assessment for their condition are often referred to psychiatrists until the time when the disease is better understood and defined. Limited integration between psychiatry and general medicine, silo mentality, restrictive diagnostic criteria, and erroneous guidelines currently contribute to diagnostic errors. Uncommon, complex, and multisystem diseases such as Lyme disease ME/CFS more often misdiagnosed as having a psychogenic etiology to their symptoms. As more sophisticated technologies emerge to visualize the brain, to demonstrate brain pathophysiology, and to quantitate mental functioning, and the causes of mental illness become better understood, the validity of many of the previously used phrases that were based upon the absence of physical findings, such as psychogenic and functional disorders, are becoming less valid. There is now an increasing amount of literature demonstrating somatopsychic and multisystem processes and the accompanying pathophysiology [57].

The diagnosis of any medical or psychiatric syndrome requires the presence of clearly defined signs and symptoms consistent with each diagnostic category. Reliance upon the total clinical exam, including an adequate history, review of systems, psychiatric assessment, and clinical judgment, is more valid than reliance upon any single laboratory or diagnostic test. When using diagnostic testing, absence of proof is never proof of absence. Although the absence of a finding in a diagnostic test may raise the suspicion of a psychiatric illness, the absence of a finding alone can never confirm the presence of a psychiatric illness. The diagnosis of a psychosomatic condition requires a causal psychodynamic explanation, and it is never a diagnosis of exclusion based upon a failure to confirm some other diagnosis. A more thorough psychiatric assessment can determine whether there is a psychodynamic and/or psychiatric pathophysiological process that would explain a symptom. The onset of a multisystem illness is rarely, if ever, associated with a psychogenic etiology. The presence of a psychiatric diagnosis does not eliminate the possibility of a comorbid, somatic diagnosis or a comorbid somatic diagnosis causing psychiatric symptoms. Many complex conditions once described as “all in your head” are immune mediated infections. These conditions may—more probably and accurately—be in the immune system and/or the microbiome [117,131,132]. 

We can learn from history. Syphilis was once a difficult to understand multisystem illness with periods of latency and a broad spectrum of presentations including both psychiatric and somatic and symptoms. Now, we are attempting to understand other multisystem, complex, interactive infectious diseases that are far more complex than syphilis. *Treponema pallidum* (syphilis) has only 22 genes. In contrast, *Borrelia burgdorferi* (the bacterium responsible for Lyme disease) has 132 genes with plasmids that allow for rapid genetic changes and interactions with other tickborne and opportunistic infections [143]. There are over one hundred other infectious agents that cause mental illnesses [57].

We always need to be alert to new and emerging diseases. We must recognize there is always some degree of medical uncertainty with any condition. Not everything is well understood or categorized. Complex diseases require complex explanations, and there needs to be recognition of varying degrees of medical uncertainty. Everything is caused by something. Nothing is caused by nothing. When clinical findings are puzzling, the ethical approach is to continue attempting to explain the symptom, search for its cause, and admit that we do not have the required knowledge to provide a cure or even complete symptom relief.

Medically unexplained symptoms, somatoform disorder, and compensation neurosis are outdated and/or inaccurate terms. Qualitative terms such as subjective, vague, and nonspecific can be used inaccurately. Conversion disorders, functional disorders, psychogenic illness, factitious disorder imposed upon another (Munchausen’s by proxy), somatic symptom disorder, psychogenic seizures, psychogenic pain, psychogenic fatigue, and delusional parasitosis can be over-diagnosed. Bodily distress disorder is highly subjective and is a scientifically unsupported and inaccurate term. Bodily distress syndrome is also highly subjective. It is dependent upon the flawed concept of medically unexplained symptoms and is a scientifically unsupported and inaccurate term. A common diagnostic pitfall with all of these terms is the risk that something unexamined or not adequately understood can result in an improper diagnosis, inadequate treatment, and inadequate financial coverage by third party payers. 

To properly understand the mind/body connection, adequate training and knowledge of general medicine, psychiatry, and the systems that link the soma and the brain are required. No one has complete knowledge of all fields of medical sciences. Not all diseases have been discovered or are properly understood, and much remains to be learned. Better education concerning the interface between medicine and psychiatry and the associated diagnostic nomenclature as well as utilizing clinical judgment and thorough assessment, exercising humility, and maintaining our roots in traditional medicine will help improve diagnostic accuracy and move both science and medicine forward.

## Figures and Tables

**Table 1:** Psychiatric diagnostic terms with misuse and abuse potential.

Term	DSM-5 Diagnosis	ICD-10 Diagnosis	ICD-11 Diagnosis
All in your head	No	No	No
Somatic symptom disorder	Yes	Yes	No
Somatoform disorder	No	No	No
Medically unexplained symptoms	No	No	No
Functional neurological symptom disorder	Yes	Yes	No
Conversion disorder	No	Yes	No
Illness anxiety disorder	Yes	No	Yes
Factitious disorder imposed upon another (Munchausen’s by proxy)	Yes	Yes	Yes
Functional disorders	No	Yes	No
Psychogenic disorders	No	Yes	No
Compensation neurosis	No	No	No
Psychogenic seizures	No	Yes	Yes
Psychogenic pain	No	Yes	No
Psychogenic fatigue	No	No	No
Delusional parasitosis	No	Yes	Yes
Subjective vs. objective	No	No	No
Non-specific or vague symptoms	No	No	No
Bodily distress disorder	No	No	Yes
Bodily distress syndrome	No	No	No

Many psychiatric terms, previous and current, have misuse and abuse potential. Some are included in the American Psychiatric Association Diagnostic and Statistical Manual 5th Edition (DSM-5), others are included in the International Classification of Diseases 10th Edition (ICD-10), others are being proposed to be included and/or dropped in the International Classification 11th Edition (ICD-11), and others have never been included in any formal diagnostic system.

## Abbreviations

The following abbreviations are used in this manuscript:

PACE	Patient graded activity and cognitive behavior therapy
IDSA	Infectious Disease Society of America
MUS	Medically Unexplained Symptoms
APA	American Psychiatric Association
DSM-5	Diagnostic and Statistical Manual of Mental Disorders, 5th Edition
ICD	International Classification of Diseases
ALPIM	Anxiety-laxity-pain-immune-mood
DSM-IV-TR	Diagnostic and Statistical Manual, Fourth Edition, Text Revision
ME/CFS	Myalgic encephalomyelitis/chronic fatigue syndrome

## References

[1] Kohlmann S., Löwe B., Shedden-Mora M.C. (2018). Health Care for Persistent Somatic Symptoms Across Europe: A Qualitative Evaluation of the EURONET-SOMA Expert Discussion. Front. Psychiatry.

[2] Orešković S. (2016). Breaking down the Silo Mentality in Global Mental Health: The New Role for the Schools of Public Health. Psychiatr. Danub..

[3] Singh Ospina N., Phillips K.A., Rodriguez-Gutierrez R., Castaneda-Guarderas A., Gionfriddo M.R., Branda M.E., Montori V.M. (2019). Eliciting the Patient’s Agenda- Secondary Analysis of Recorded Clinical Encounters. J. Gen. Intern. Med..

[4] Rubio D.M., Schoenbaum E.E., Lee L.S., Schteingart D.E., Marantz P.R., Anderson K.E., Platt L.D., Baez A., Esposito K. (2010). Defining translational research: Implications for training. Acad. Med..

[5] Second National Conference on Serological diagnosis of Lyme Disease Association of State and Territorial Public Health Laboratory Directors. Dearborn, Michigan, USA in 1994. http://www.actionlyme.org/DEARBORN_PDF.pdf.

[6] Boyd C.M., Darer J., Boult C., Fried L.P., Boult L., Wu A.W. (2005). Clinical practice guidelines and quality of care for older patients with multiple comorbid diseases: Implications for pay for performance. JAMA.

[7] Hurwitz B. (1995). Clinical guidelines and the law: Advice, guidance or regulation?. J. Eval. Clin. Pract..

[8] Johnson L., Stricker R. (2004). Treatment of Lyme disease: A medicolegal assessment. Expert Rev. Anti-infec. Ther..

[9] (1990). Wilson *v*. Blue Cross of Southern California, 271 Cal. Rptr. 876. https://www.courtlistener.com/opinion/2159785/wilson-v-blue-cross-of-so-california/.

[10] Johnson L. (2019). 2019 Chart Book—MyLymeData Registry; (Phase 1 April 27, 2017. Sample 3,903). Figshare. Preprint.

[11] Grupp H., Kaufmann C., König H.H., Bleibler F., Wild B., Szecsenyi J., Herzog W., Schellberg D., Schäfert R., Konnopka A. (2017). Excess costs from functional somatic syndromes in Germany—An analysis using entropy balancing. J. Psychosom. Res..

[12] Cutshaw C.A., Woolhandler S., Himmelstein D.U., Robertson C. (2016). Medical Causes and Consequences of Home Foreclosures. Int. J. Health Serv..

[13] Adrion E.R., Aucott J., Lemke K.W., Weiner J.P. (2015). Health care costs, utilization and patterns of care following Lyme disease. PLoS ONE.

[14] Brownell A.K.W., Atkins C., Whiteley A., Woollard R.F., Kornelsen J. (2016). Clinical practitioners’ views on the management physical symptoms (MUPS): A qualitative study. BMJ Open.

[15] Hartman O.T.C., Hassink-Franke L.J., Lucassen P.L., Van Spaendonck K.P., Van Weel C. (2009). Explanation and relations. how do general practitioners deal with patients with persistent medically unexplained symptoms: A focus group study. BMC Fam. Pract..

[16] McAndrew L.M., Philips L.A., Helmer D.A., Maestro K., Engel C.C., Greenberg L.M., Anastasides N., Quigley K.S. (2017). High healthcare utilization near the onset of medically unexplained symptoms. J. Psychosom. Res..

[17] Weintraub P. (2008). Cure Unknown: Inside the Lyme Epidemic.

[18] Hilfiger A. (2016). Bite Me: How Lyme Disease Stole My Childhood, Made Me Crazy, and Almost Killed Me.

[19] Dusenbery M. (2018). Doing Harm.

[20] Kraaijeveld H. (2017). The Ultimate Lyme Test; Julia’s Story. https://on-lyme.org/en/sufferers/lyme-stories/item/260-both-blessed-and-cursed-julia-s-story.

[21] Vincent I. (2016). How the Pope Healed Me. New York Post. https://nypost.com/2016/03/27/how-the-pope-healed-me/.

[22] Staff The Lyme Wars: Investigating a Public Health Crisis. NBC Chicago. https://www.nbcchicago.com/investigations/national-investigations/Lyme-Disease-Medicine-Symptoms-Diagnose-Tick-Bite-Debate-Doctor-CDC-Bullseye-Rash-451834613.html.

[23] Tanner C. (2019). Woman Eventually Diagnosed with Lyme Disease Was Sectioned for Months and Told Her Symptoms Were ‘All in Her Head.’ I News. https://inews.co.uk/news/real-life/woman-eventually-diagnosed-with-lyme-disease-was-sectioned-for-months-and-told-her-symptoms-were-all-in-her-head/?fbclid=IwAR08m3SKXrVwWn2Js6hhRfOeFxOz-UsZQ1-vIls_E7MTs0z89xZyVLZoXEw.

[24] Yao J., Lv D., Chen W. (2018). Multiple Myeloma, Misdiagnosed as Somatic Symptom Disorder: A Case Report. Front. Psychiatry.

[25] Poloni N., Ielmini M., Caselli I., Ceccon F., Bianchi L., Isella C., Callegari C. (2018). Medically Unexplained Physical Symptoms in Hospitalized Patients: A 9-Year Retrospective Observational Study. Front. Psychiatry.

[26] Lacourt T.E., Houtveen J.H., Smeets H.M., Lipovsky M.M., van Doornen L.J. (2013). Infection load as a predisposing factor for somatoform disorders: Evidence from a Dutch General Practice Registry. Psychosom. Med..

[27] Kim J.Y., Ko I., Kim M.S., Yu M.S., Cho B.J., Kim D.K. (2019). Association of Chronic Rhinosinusitis with Depression and Anxiety in a Nationwide Insurance Population. JAMA Otolaryngol. Head Neck Surg..

[28] Labrie V., Brundin L. (2018). Harbingers of Mental Disease-Infections Associated with an Increased Risk for Neuropsychiatric Illness in Children. JAMA Psychiatry.

[29] Pederson C.L., Brookings J.B. (2018). Suicide Risk Linked with Perceived Burdensomeness in Postural Tachycardia Syndrome. J. Health Sci. Educ..

[30] Smith G.C., Pell J.P. (2003). Parachute use to prevent death and major trauma related to gravitational challenge: Systematic review of randomised controlled trials. BMJ.

[31] Committee on the Diagnostic Criteria for Myalgic Encephalomyelitis/Chronic Fatigue Syndrome, Board on the Health of Select Populations, Institute of Medicine (2015). Beyond Myalgic Encephalomyelitis/Chronic Fatigue Syndrome: Redefining an Illness.

[32] Henderson T. (2015). The Role of Antiviral Therapy in Chronic Fatigue Treatment. Psychiatric Advisor. https://www.psychiatryadvisor.com/home/opinion/the-role-of-antiviral-therapy-in-chronic-fatigue-treatment/.

[33] Smith B., Nelson H.D., Haney E., Pappas M., Daeges M., Wasson N., McDonagh M. (2014). Diagnosis and Treatment of Myalgic Encephalomyelitis/Chronic Fatigue Syndrome.

[34] Twisk F.N., Maes M. (2009). A review on cognitive behavorial therapy (CBT) and graded exercise therapy (GET) in myalgic encephalomyelitis (ME) / chronic fatigue syndrome (CFS). Neuro Endocrinol. Lett..

[35] Torjesen I. (2018). Pressure grows on Lancet to review “flawed” PACE trial. BMJ.

[36] Rehmeyer J. Bad Science Misled Millions with Chronic Fatigue Syndrome. Here’s how We Fought Back. https://www.statnews.com/2016/09/21/chronic-fatigue-syndrome-pace-trial/.

[37] White P.D., Goldsmith K.A., Johnson A.L., Potts L., Walwyn R., DeCesare J.C., Baber H.L., Burgess M., Clark L.V., Cox D.L. (2011). Comparison of adaptive pacing therapy, cognitive behaviour therapy, graded exercise therapy, and specialist medical care for chronic fatigue syndrome (PACE): A randomized trial. Lancet.

[38] Tuller D. Trial by Error: Some Good News on Cochrane. Virology Blog. http://www.virology.ws/2018/12/03/trial-by-error-some-good-news-on-cochrane.

[39] Friedman K.J. (2019). Advances in ME/CFS: Past, Present, and Future. Front. Pediatr..

[40] Wormser G.P., Dattwyler R.J., Shapiro E.D., Halperin J.J., Steere A.C., Klempner M.S., Krause P.J., Bakken J.S., Strle F., Stanek G. (2006). The clinical assessment, treatment, and prevention of Lyme disease, human granulocytic anaplasmosis, and babesiosis: Clinical practice guidelines by the Infectious Diseases Society of America. Clin. Infect. Dis..

[41] Johnson L., Stricker R.B. (2010). The Infectious Diseases Society of America Lyme guidelines: A cautionary tale about the development of clinical practice guidelines. Philos. Ethics Humanit. Med..

[42] Delong A.K., Blossom B., Maloney E.L., Phillips S.E. (2012). Antibiotic retreatment of Lyme disease in patients with persistent symptoms: A biostatistical review of randomized, placebo-controlled, clinical trials. Contemp. Clin. Trials.

[43] Cameron D.J. (2006). Generalizability in two clinical trials of Lyme disease. Epidemiol. Perspect. Innov..

[44] Khan A.R., Khan S., Zimmerman V., Baddour L.M., Tleyjeh I.M. (2010). Quality and strength of evidence of the Infectious Diseases Society of America clinical practice guidelines. Clin. Infect. Dis..

[45] Lee D.H., Vielemeyer O. (2011). Analysis of overall level of evidence behind Infectious Diseases Society of America practice guidelines. Arch. Intern. Med..

[46] Lenzer J., Hoffman J.R., Furberg C.D., Ioannidis J.P., Guideline Panel Review Working Group (2013). Ensuring the integrity of clinical practice guidelines: A tool for protecting patients. BMJ.

[47] Davidsson M. (2018). The Financial Implications of a Well-Hidden and Ignored Chronic Lyme Disease Pandemic. Healthcare (Basel).

[48] Graham R., Mancher M., Miller Wolman D., Greenfield S., Steinberg E. (2011). Institute of Medicine (US) Committee on Standards for Developing Trustworthy Clinical Practice Guidelines.

[49] Ewald P., Liegner K.B. (2015). The Crucible of Chronic Lyme Disease: Collected Writings & Associated Materials.

[50] Bransfield R.C., Cook M.J., Bransfield D.R. (2019). Proposed Lyme Disease Guidelines and Psychiatric Illnesses. Healthcare.

[51] Johnson L., Maloney E. The Ad Hoc Patient and Physician Coalition Comments of the IDSA Proposed Lyme Guidelines 8 August 2019. https://www.lymedisease.org/wp-content/uploads/2019/08/Ad-Hoc-Patient-Physician-Coalition-Comments.pdf.

[52] Lyme Disease Guidelines. www.lymediseaseguidelines.org.

[53] Blake L., Davies V., Conn R., Davie M. (2018). Medically Unexplained Symptoms (Mus) In Children And Young People: A Guide To Assessing And Managing Patients Under The Age Of 18 Who Are Referred To Secondary Care. https://paedmhassoc.files.wordpress.com/2018/11/mus-guide-with-leaflet-nov-2018.pdf.

[54] American Psychiatric Association (2013). Diagnostic and Statistical Manual of Mental Disorders.

[55] International Classification of Diseases 11th Revision. https://icd.who.int/en/.

[56] Coplan J., Singh D., Gopinath S., Mathew S.J., Bulbena A. (2015). A Novel Anxiety and Affective Spectrum Disorder of Mind and Body-The ALPIM (Anxiety-Laxity-Pain-Immune-Mood) Syndrome: A Preliminary Report. J. Neuropsychiatry Clin. Neurosci..

[57] Bransfield R.C. (2018). Neuropsychiatric Lyme Borreliosis: An Overview with a Focus on a Specialty Psychiatrist’s Clinical Practice. Healthcare.

[58] Bransfield R.C. (2018). Aggressiveness, violence, homicidality, homicide, and Lyme disease. Neuropsychiatr. Dis. Treat..

[59] Bransfield R.C. (2017). Suicide and Lyme and associated diseases. Neuropsychiatr. Dis. Treat..

[60] Bransfield R.C., Wulfman J.S., Harvey W.T., Usman A.I. (2008). The association between tick-borne infections, Lyme borreliosis and autism spectrum disorders. Med. Hypotheses.

[61] Bransfield R.C. (2009). Preventable cases of autism: The relationship between chronic infectious diseases and neurological outcome. Pediatr. Health.

[62] Fallon B.A., Schwartzberg M., Bransfield R., Zimmerman B., Scotti A., Weber C.A., Liebowitz M.R. (1995). Late-stage neuropsychiatric Lyme borreliosis. Differential diagnosis and treatment. Psychosomatics.

[63] Socrates, Quotes, Quotable Quote, GoodReads. https://www.goodreads.com/quotes/132404-the-beginning-of-wisdom-is-the-definition-of-terms.

[64] Bransfield R.C. (1999). Mental Health. http://www.mentalhealthandillness.com/health.html.

[65] Department of Health and Human Services (1999). Mental Health: A Report of the Surgeon General. https://profiles.nlm.nih.gov/ps/access/NNBBHS.pdf.

[66] Abu-Asab M.S., Chaouchi M., Alesci S., Galli S., Laassri M., Cheema A.K., Atouf F., VanMeter J., Amri H. (2011). Biomarkers in the age of omics: Time for a systems biology approach. OMICS.

[67] Bransfield R.C. (2019). Somatopsychic, Psychosomatic or Multisystem Illness?.

[68] Even N., Devaud J.M., Barron A.B. (2012). General Stress Responses in the Honey Bee. Insects.

[69] Wang J., Rao H., Wetmore G.S., Furlan P.M., Korczykowski M., Dinges D.F., Detre J.A. (2005). Perfusion functional MRI reveals cerebral blood flow pattern under psychological stress. Proc. Natl. Acad. Sci. USA.

[70] Arnsten A.F. (2009). Stress signalling pathways that impair prefrontal cortex structure and function. Nat. Rev. Neurosci..

[71] Halaris A. (2018). Psychocardiology: Understanding heart brain connection. Psychiatric Times.

[72] Zamani M., Alizadeh-Tabari S., Zamani V. (2019). Systematic review with meta-analysis: The prevalence of anxiety and depression in patients with irritable bowel syndrome. Aliment. Pharmacol. Ther..

[73] Roszkowska A., Pawlicka M., Mroczek A., Bałabuszek K., Nieradko-Iwanicka B. (2019). Non-Celiac Gluten Sensitivity: A Review. Medicina.

[74] Bransfield R.C. (1999). Microbes and mental Illness. https://www.mentalhealthandillness.com/Articles/MicrobesAndMentalIllness.htm.

[75] Peer-Reviewed Evidence of Persistence of Lyme Disease Spirochete Borrelia burgdorferi and Tick-Borne Diseases: Neuropsychiatric Symptoms and Lyme/Tick-Borne Diseases. https://www.ilads.org/wp-content/uploads/2018/07/CLDList-ILADS.pdf.

[76] Van Mierlo H.C., Schot A., Boks M.P.M., de Witte L.D. (2019). The association between schizophrenia and the immune system: Review of the evidence from unbiased ‘omic-studies’. Schizophr. Res..

[77] Fenchel D., Levkovitz Y., Kotler M. (2017). [BIPOLAR DISORDER AS A MULTI-SYSTEM ILLNESS]. Harefuah.

[78] Guest P.C. (2019). Insulin Resistance in Schizophrenia. Adv. Exp. Med. Biol..

[79] Dickerson F., Severance E., Yolken R. (2017). The microbiome, immunity, and schizophrenia and bipolar disorder. Brain Behav. Immun..

[80] Bhise V., Rajan S.S., Sittig D.F., Morgan R.O., Chaudhary P., Singh H. (2018). Defining and Measuring Diagnostic Uncertainty in Medicine: A Systematic Review. J. Gen. Intern. Med..

[81] Defense.gov News Transcript: DoD News Briefing—Secretary Rumsfeld and Gen. Myers, United States Department of Defense (defense.gov). http://archive.defense.gov/Transcripts/Transcript.aspx?TranscriptID=2636.

[82] American Psychiatric Association (2000). Diagnostic and Statistical Manual of Mental Disorders.

[83] U.S. Department of Health and Human Services, National Institutes of Health, National Center for Advancing Transitional Sciences, Genetics and Rare Diseases Information Center, Conversion Disorder. https://rarediseases.info.nih.gov/diseases/6191/conversion-disorder#ref_2557.

[84] McMullan R.D., Berle D., Arnáez S., Starcevic V. (2019). The relationships between health anxiety, online health information seeking, and cyberchondria: Systematic review and meta-analysis. J. Affect. Disord..

[85] Sherr V.T. (2005). Munchausen’s syndrome by proxy and Lyme disease: Medical misogyny or diagnostic mystery?. Med. Hypotheses.

[86] Meijer S. A New Stolen Generation. 27 May 2018. Lymedisease.org. https://www.lymedisease.org/child-lyme-netherlands/.

[87] ICDData.com. https://www.icd10data.com/ICD10CM/Codes/F01-F99/F40-F48/F45-/F45.8.

[88] Mendelson G. (1985). “Compensation neurosis”. An invalid diagnosis. Med. J. Aust..

[89] Tolchin B., Martino S., Hirsch L.J. (2019). Treatment of Patients with Psychogenic Nonepileptic Attacks. JAMA.

[90] Yogarajah M., Child R., Agrawal N., Cope S., Edwards M., Mula M. (2018). Functional seizures: An evaluation of the attitudes of general practitioners local to a tertiary neuroscience service in London. Epilepsia Open.

[91] Smith S.J.M. (2005). EEG in the diagnosis, classification, and management of patients with epilepsy. J. Neurol. Neurosurg. Psychiatry.

[92] Danilov A.B., Isagilyan E.D., Mackaschova E.S. (2018). [Psychogenic pain]. Zh Nevrol Psikhiatr Im S S Korsakova.

[93] Elman I., Borsook D. (2018). Threat Response System: Parallel Brain Processes in Pain vis-à-vis Fear and Anxiety. Front. Psychiatry.

[94] Arnow B.A., Hunkeler E.M., Blasey C.M., Lee J., Constantino M.J., Fireman B., Kraemer H.C., Dea R., Robinson R., Hayward C. (2006). Comorbid depression, chronic pain, and disability in primary care. Psychosom. Med..

[95] Robinson M.J., Edwards S.E., Iyengar S., Bymaster F., Clark M., Katon W. (2009). Depression and pain. Front. Biosci. (Landmark Ed.).

[96] Van der Hulst M., Vollenbroek-Hutten M.M., Rietman J.S., Hermens H.J. (2010). Lumbar and abdominal muscle activity during walking in subjects with chronic low back pain: Support of the “guarding” hypothesis?. J. Electromyogr. Kinesiol..

[97] Van der Hulst M., Vollenbroek-Hutten M.M., Rietman J.S., Schaake L., Groothuis-Oudshoorn K.G., Hermens H.J. (2010). Back muscle activation patterns in chronic low back pain during walking: A “guarding” hypothesis. Clin. J. Pain.

[98] Bates D., Schultheis B.C., Hanes M.C., Jolly S.M., Chakravarthy K.V., Deer T.R., Levy R.M., Hunter C.W. (2019). A Comprehensive Algorithm for Management of Neuropathic Pain. Pain Med..

[99] Gierthmühlen J., Schneider U., Seemann M., Freitag-Wolf S., Maihöfner C., Enax-Krumova E.K., Azad S.C., Üçeyler N., Birklein F., Maier C. (2019). Can self-reported pain characteristics and bedside test be used for the assessment of pain mechanisms? An analysis of results of neuropathic pain questionnaires and quantitative sensory testing. Pain.

[100] Aich A., Afrin L.B., Gupta K. (2015). Mast Cell-Mediated Mechanisms of Nociception. Int. J. Mol. Sci..

[101] Complex Regional Pain Syndrome Fact Sheet National Institute of Neurological Disorders and Stroke. https://www.ninds.nih.gov/Disorders/Patient-Caregiver-Education/Fact-Sheets/Complex-Regional-Pain-Syndrome-Fact-Sheet.

[102] Hofbauer R.K., Olausson H.W., Bushnell M.C. (2006). Thermal and tactile sensory deficits and allodynia in a nerve-injured patient: A multimodal psychophysical and functional magnetic resonance imaging study. Clin. J. Pain.

[103] Freudenmann R.W., Lepping P. (2009). Delusional infestation. Clin. Microbiol. Rev..

[104] ICD-10. https://www.icd10data.com/ICD10CM/Codes/F01-F99/F01-F09/F06-/F06.2.

[105] Vila-Rodriguez F., Macewan B.G. (2008). Delusional parasitosis facilitated by web-based dissemination. Am. J. Psychiatry.

[106] Amin O. (2015). Disseminated dermatological symptoms in chronic cases of Neurocutaneous Syndrome (NCS) or Morgellons. JMM Case Rep..

[107] Middelveen M.J., Fesler M.C., Stricker R.B. (2018). History of Morgellons disease: From delusion to definition. Clin. Cosmet. Investig. Dermatol..

[108] Middelveen M.J., Stricker R.B. (2016). Morgellons disease: A filamentous borrelial dermatitis. Int. J. Gen. Med..

[109] Baker C.J., Charini W.A., Lantos P.M., Medoff G., Moro M.H., Mushatt D.M., Parsonnet J., Sanders J.W. Final Report of the Lyme Disease Review Panel of the Infectious Diseases Society of America (IDSA). https://www.idsociety.org/globalassets/idsa/topics-of-interest/lyme/idsalymediseasefinalreport.pdf.

[110] Klempner M.S., Hum L.T., Evans J., Schmid C.H., Johnson G.M., Trevino R.P., Norton D., Levy L., Wall D., McCall J. (2001). Two controlled trials of antibiotic treatment in patients with persistent symptoms and a history of Lyme disease. N. Engl. J. Med..

[111] Cook M.J., Puri B.K. (2017). Application of Bayesian decision-making to laboratory testing for Lyme disease and comparison with testing for HIV. Int. J. Gen. Med..

[112] Bransfield R., Brand S., Sherr V. (2001). Treatment of patients with persistent symptoms and a history of Lyme disease. N. Engl. J. Med..

[113] Lewis R. (2017). Improving diagnostic pathways for patients with vague symptoms Executive summary Accelerate, Coordinate, evaluate (ACE) Programme an early diagnosis of cancer initiative supported by: NHS England, Cancer Research UK and Macmillan Cancer Support, ACE Vague Symptoms Pathway Cluster. https://www.cancerresearchuk.org/sites/default/files/improving_diagnostic_pathways_for_patients_with_vague_symptoms_.pdf.

[114] Krupp L.B., LaRocca N.G., Muir-Nash J., Steinberg A.D. (1989). The fatigue severity scale. Application to patients with multiple sclerosis and systemic lupus erythematosus. Arch. Neurol..

[115] Williamson A., Hoggart B. (2005). Pain: A review of three commonly used pain rating scales. J. Clin. Nurs..

[116] Nasrallah I., Dubroff J. (2013). An overview of PET neuroimaging. Semin. Nucl. Med..

[117] Bransfield R.C. (2012). The psychoimmunology of Lyme/tick-borne diseases and its association with neuropsychiatric symptoms. Open Neurol. J..

[118] Morris G., Anderson G., Galecki P., Berk M., Maes M. (2013). A narrative review on the similarities and dissimilarities between myalgic encephalomyelitis/chronic fatigue syndrome (ME/CFS) and sickness behavior. BMC Med..

[119] Ivbijaro G., Goldberg D. (2013). Bodily distress syndrome (BDS): The evolution from medically unexplained symptoms (MUS). Ment. Health Fam. Med..

[120] Budtz-Lilly A., Schröder A., Rask M.T., Fink P., Vestergaard M., Rosendal M. (2015). Bodily distress syndrome: A new diagnosis for functional disorders in primary care?. BMC Fam. Pract..

[121] Dx Revision Watch. https://dxrevisionwatch.files.wordpress.com/2018/07/comparison-of-ssd-bdd-bds-bss-in-classification-systems-v1.pdf.

[122] ICD-11 for Mortality and Morbidity Statistics (Version: 04/2019). https://icd.who.int/browse11/l-m/en#/http%3a%2f%2fid.who.int%2ficd%2fentity%2f794195577.

[123] Gureje O., Reed G.M. (2016). Bodily distress disorder in ICD-11: Problems and prospects. World Psychiatry.

[124] Fink P., Toft T., Hansen M.S., Ørnbøl E., Olesen F. (2007). Symptoms and syndromes of bodily distress: An exploratory study of 978 internal medical, neurological, and primary care patients. Psychosom. Med..

[125] O’Leary D. (2018). Bodily Distress Syndrome: Concerns About Scientific Credibility in Research and Implementation. J. Biol. Phys. Chem..

[126] Rask M.T., Ørnbøl E., Rosendal M., Fink P. (2017). Long-Term Outcome of Bodily Distress Syndrome in Primary Care: A Follow-Up Study on Health Care Costs, Work Disability, and Self-Rated Health. Psychosom. Med..

[127] Frances A.. https://twitter.com/allenfrancesmd/status/949274595357376514?lang=en.

[128] Herzog J.I., Schmahl C. (2018). Adverse Childhood Experiences and the Consequences on Neurobiological, Psychosocial, and Somatic Conditions Across the Lifespan. Front. Psychiatry.

[129] Beutel M.E., Tibubos A.N., Klein E.M., Schmutzer G., Reiner I., Kocalevent R.D., Brähler E. (2017). Childhood adversities and distress—The role of resilience in a representative sample. PLoS ONE.

[130] McCarron P., Gunnell D., Harrison G.L., Okasha M., Davey Smith G. (2003). Temperament in young adulthood and later mortality: Prospective observational study. J. Epidemiol. Community Health.

[131] Pape K., Tamouza R., Leboyer M., Zipp F. (2019). Immunoneuropsychiatry—Novel perspectives on brain disorders. Nat. Rev. Neurol..

[132] Kayser M.S., Dalmau J. (2011). The emerging link between autoimmune disorders and neuropsychiatric disease. J. Neuropsychiatry Clin. Neurosci..

[133] Fallon B.A., Keilp J.G., Corbera K.M., Petkova E., Britton C.B., Dwyer E., Slavov I., Cheng J., Dobkin J., Nelson D.R. (2008). A randomized, placebo-controlled trial of repeated IV antibiotic therapy for Lyme encephalopathy. Neurology.

[134] Penner I.K., Paul F. (2017). Fatigue as a symptom or comorbidity of neurological diseases. Nat. Rev. Neurol..

[135] Aaronson L.S., Teel C.S., Cassmeyer V., Neuberger G.B., Pallikkathayil L., Pierce J., Press A.N., Williams P.D., Wingate A. (1999). Defining and measuring fatigue. Image J. Nurs. Sch..

[136] Sears C. IDSA response to the Tick-borne Disease Working Group 2018 Report to Congress, Access section. https://www.idsociety.org/globalassets/idsa/policy--advocacy/current_topics_and_issues/emerging_infections_and_biothreats/agency-efforts/112618-idsa-comments-on-tickborne-disease-working-group-report.pdf.

[137] Peri F., Nisticò D., Morabito G., Occhipinti A., Ventura A., Barbi E., Cozzi G. (2019). Somatic symptom disorder should be suspected in children with alleged chronic Lyme disease. Eur. J. Pediatr..

[138] William Osler Quotes Brainy Quote. https://www.brainyquote.com/authors/william_osler.

[139] Institute of Medicine Committee on Quality of Health Care in America (2001) (2001). Crossing the Quality Chasm: A New Health System for the 21st Century.

[140] Nussbaum A. (2016). The Finest Traditions of My Calling: One Physician’s Search for the Renewal of Medicine.

[141] Shannon M.T. (2011). Please Hear What I’m Not Saying: The Art of Listening in the Clinical Encounter. Perm. J..

[142] (2019). Miles Sibley: Evidence-Based Practice—A Double standard?. BMJ Opinion.

[143] Brisson D., Drecktrah D., Eggers C.H., Samuels D.S. (2012). Genetics of Borrelia burgdorferi. Annu. Rev. Genet..

